# Diathermy and bone sawing are high aerosol yield procedures: observations from a sterile laminar flow environment

**DOI:** 10.1302/2046-3758.1210.BJR-2023-0028.R1

**Published:** 2023-10-10

**Authors:** Victoria Hamilton, Sadiyah Sheikh, Alicja Szczepanska, Nick Maskell, Fergus Hamilton, Jonathan P. Reid, Bryan R. Bzdek, James R. D. Murray

**Affiliations:** 1 School of Chemistry, University of Bristol, Bristol, UK; 2 Bristol Medical School, University of Bristol, Bristol, UK; 3 Avon Orthopaedic Centre, Southmead Hospital, North Bristol NHS Trust, Bristol, UK

**Keywords:** Aerosol generating procedure, Orthopaedic, COVID-19, diathermy, orthopaedic procedures, knee, hip, saline, osteotomes, blood, personal protective equipment (PPE), Clinicians, shoulder

## Abstract

**Aims:**

Orthopaedic surgery uses many varied instruments with high-speed, high-impact, thermal energy and sometimes heavy instruments, all of which potentially result in aerosolization of contaminated blood, tissue, and bone, raising concerns for clinicians’ health. This study quantifies the aerosol exposure by measuring the number and size distribution of the particles reaching the lead surgeon during key orthopaedic operations.

**Methods:**

The aerosol yield from 17 orthopaedic open surgeries (on the knee, hip, and shoulder) was recorded at the position of the lead surgeon using an Aerodynamic Particle Sizer (APS; 0.5 to 20 μm diameter particles) sampling at 1 s time resolution. Through timestamping, detected aerosol was attributed to specific procedures.

**Results:**

Diathermy (electrocautery) and oscillating bone saw use had a high aerosol yield (> 100 particles detected per s) consistent with high exposure to aerosol in the respirable range (< 5 µm) for the lead surgeon. Pulsed lavage, reaming, osteotome use, and jig application/removal were medium aerosol yield (10 to 100 particles s^-1^). However, pulsed lavage aerosol was largely attributed to the saline jet, osteotome use was always brief, and jig application/removal had a large variability in the associated aerosol yield. Suctioning (with/without saline irrigation) had a low aerosol yield (< 10 particles s^-1^). Most surprisingly, other high-speed procedures, such as drilling and screwing, had low aerosol yields.

**Conclusion:**

This work suggests that additional precautions should be recommended for diathermy and bone sawing, such as enhanced personal protective equipment or the use of suction devices to reduce exposure.

Cite this article: *Bone Joint Res* 2023;12(10):636–643.

## Article focus

- Quantification of aerosol exposure to lead surgeon during routine open orthopaedic surgery.

## Key messages

- A novel sterile sampling setup was used to detect aerosol throughout surgery.
- A high aerosol yield within the respirable size range was observed consistently during diathermy and bone saw use.
- Additional precautions should be recommended for these procedures.

## Strengths and limitations

- Two highly sensitive aerosol particle size detectors were used concurrently to detect aerosol at the site of the lead surgeon throughout 17 common orthopaedic procedures.
- Control experiments were performed during simulated phantom surgery.
- Placement of detector openings was variable between surgeries, and directionality of aerosol could not be determined.

## Introduction

The COVID-19 respiratory disease pandemic has placed substantial stress on healthcare systems. The SARS-CoV-2 virus is transmitted from an infected individual through fomites, droplets, and aerosols.^1^ Transmission is largely attributed to droplets, but the contribution of aerosols to transmission is now widely accepted.^2,3^ The World Health Organization (WHO) defines aerosols as particles below ≤ 5 µm.^4^ This definition describes the size threshold for penetration to the deepest part of the respiratory tract, but particles ≤ 100 µm in diameter are inhalable and capable of suspension in air.^5^

At the start of the pandemic, any surgical procedure involving a high-speed instrument was classified as an aerosol-generating procedure (AGP). Reviews listed the use of thermal, high-speed, heavy, and high-impact instruments as AGPs, concluding that these common procedures in orthopaedic surgery result in a high possibility of aerosolization of biological material which may contain viable virus.^6-8^ Therefore, orthopaedic surgery was classified as a high-risk speciality. Protective protocols were recommended, including high-level personal protective equipment (PPE), increased operating theatre air changes, and fallow times. Unfortunately, these additional requirements resulted in substantial delays to patient care, difficulty operating in the requisite PPE for long periods of time with poor visibility, and increased risk of desterilization – vital to avoid in implant surgery. Without appropriate evidence to categorize high- and low-risk procedures, PPE was distributed on a common sense approach, considering ward versus surgical use, and resulted in environmental waste without appropriate protection for healthcare workers.

The WHO now specifies high-risk AGPs in healthcare settings as “aerosol-generating and consistently associated with an increased risk of pathogen transmission”.^4^ Accordingly, the recent UK government AGP review only lists high-speed cutting, identifying “no evidence of appropriate strength or quality” for categorizing drilling, diathermy, irrigation, manual sawing, or pulsed lavage.^9^ In this study, aerosol yield was recorded from the position of the lead surgeon (JRDM, closest to aerosol generation) in a range of orthopaedic operations (hip, knee, and shoulder), aiming to define the extent of the likely aerosol generation from specific procedures within an operation and thus the exposure risk of the lead surgeon.

## Methods

### Data collection

All reported aerosol measurements were obtained using an Aerodynamic Particle Sizer (APS) (TSI model 3321, 0.5 to 20 μm diameter particles; TSI, USA). The APS measures at a sampling flow rate of 1 l min^-1^ (with 4 l min^-1^ sheath flow), and sampling time resolution is 1 s. Measured aerosols were assigned into 52 size-resolved bins. Aerosol was sampled continuously from onset of the surgery until suturing. Measurements were made in an operating theatre under laminar flow (Howorth Exflow 32 Evolution; Howorth Air Technology, UK), affording an environment with nearly zero background aerosol concentration in the APS size range, allowing unambiguous attribution of specific activities to aerosol generation.^10^

**Fig. 1:**
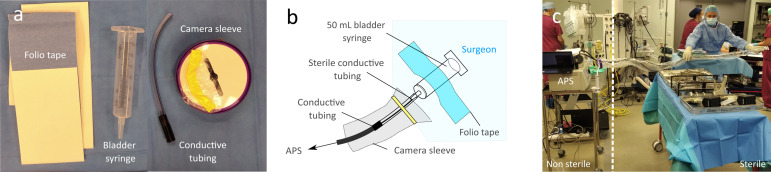
The sterile setup for aerosol detection during surgery. a) Sterile components: folio tape, 50 ml bladder syringe (plunger discarded), 20 cm sterile conductive tubing, and camera sleeve. b) Labelled schematic of assemblage. c) Setup allows an approximately 2 m sterile range with the non-sterile region attaching to the particle detector (Aerodynamic Particle Sizer (APS)) outside of laminar flow region.

To ensure complete sterility, a novel sampling setup was assembled from sterile components (Figure 1a) in the operating theatre by the surgeon (JRDM) or surgical assistants before each surgery. Figure 1b shows the final assemblage. Sterile conductive universal suction connecting tubing (Nutwell Medical, UK) was cut to 20 cm and connected to a large (50 ml) bladder syringe tip (plunger discarded). An endoscopic camera sleeve (Ring Frame Camera Sleeve; Purple Surgical, UK) was then secured to the assemblage. The conductive sterile tubing was then connected to 2 m length of non-sterile ¼” conductive silicone sampling tubing (TSI), which was then encased within the sterile sleeve by retracting the sleeve out of the operative canopy (in the same manner as dressing an arthroscope) until reaching the APS (Figure 1c). Folio tape (sterile drape securing adhesive tape – non-proprietary) secured the syringe to the surgeon (side of the lower chest, closest to the operative field) with the open end directed towards the surgery site, enabling sterile detection of aerosol during orthopaedic surgery as close to the source as practicable without interfering with surgical dexterity.

Aerosol cannot be detected at source during surgery due to the need for total visibility and sterility of the surgical site. The syringes were instead attached to the lead surgeon, resulting in a detection distance ranging from approximately 20 to 50 cm. Due to the variable detection distance, aerosol measurements cannot rationally be discussed in terms of total particle concentration (cm^-3^) as this value will decay proportionately with dispersion over increasing distance from source, further complicated by laminar flow and possible directionality of the generated aerosol plume. The lead surgeon is typically closest to the surgical site and thus most exposed to aerosol. Therefore, aerosol yield is discussed in terms of the number of aerosol particles detected per second (count (s^-1^)), which directly relates to lead surgeon exposure and allows different procedures to be compared to assess relative risk.

### Data processing

Background aerosol measurements were acquired in triplicate by taking 30 s intervals at approximately the beginning, midpoint, and end of each surgery. The mean background aerosol yield during the 17 open surgeries was 0.7 s^-1^, a consequence of the presence of the surgical team and patient (vs an empty room for undisturbed laminar flow).

**Fig. 2:**
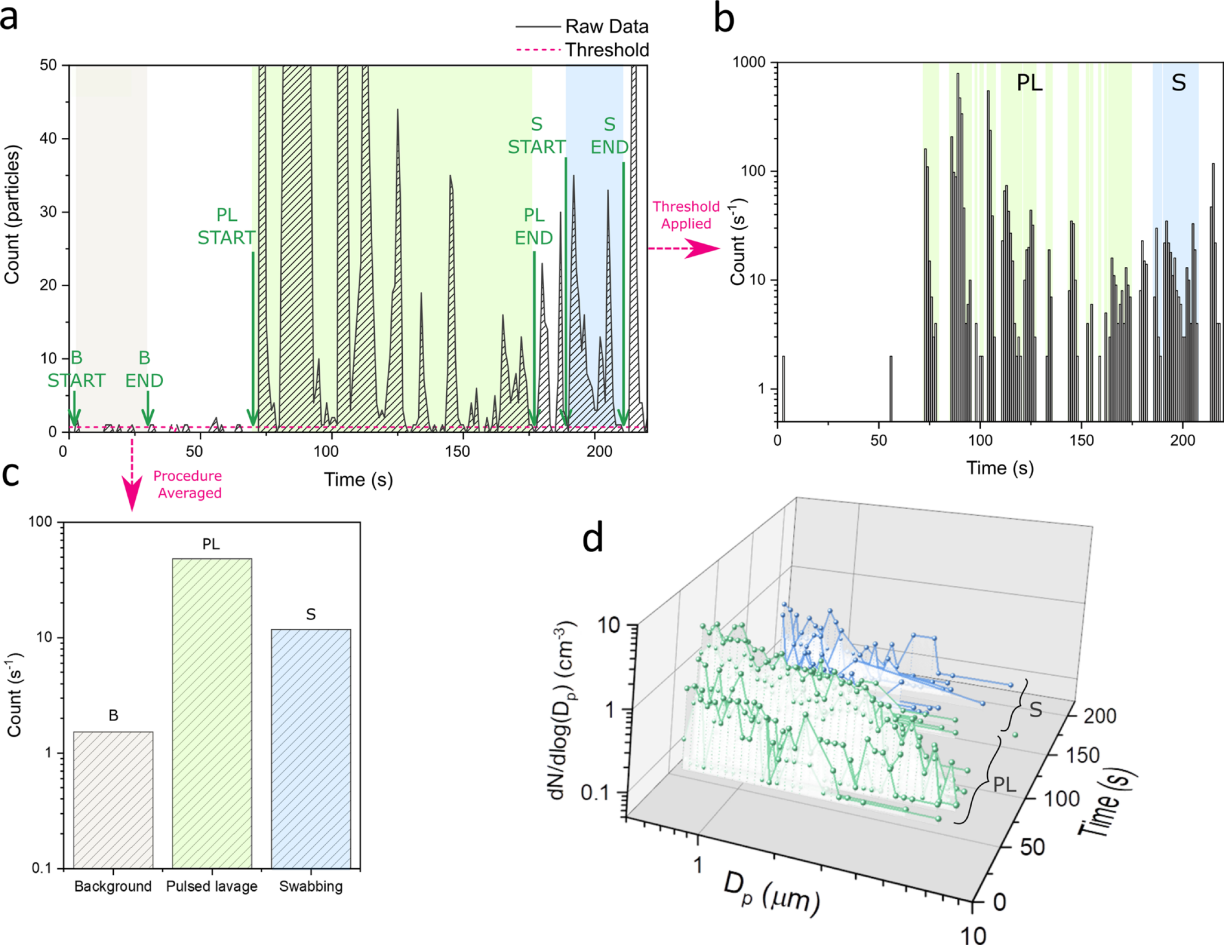
Example data analysis: background (B, grey), pulsed lavage (PL, green), and swabbing (S, blue). a) Particle count against time (black line), events (green arrows), and mean background threshold (pink dash). b) Data below threshold removed, shown as 1 s bins on a log scale. c) Average aerosol yield for each procedure. d) Size distributions: mean number concentration of particles normalized by bin width (dN/dlog(D_p_)) in each size bin (D_p_) at each second.

‘Events’ where a procedure was performed during surgery were manually time-stamped, allowing assignment of detected aerosol to particular operative tasks, e.g. drilling or reaming. The data were analyzed as shown for the example events (background, pulsed lavage, and swabbing) in Figure 2. Figure 2a shows the raw data, the number of particles detected against time with time-stamped events. Data points below the mean background threshold were removed and the aerosol yield from each event can be clearly visualized in Figure 2b by plotting the detected particle count per second on a log scale. Finally, averaging the count over the total seconds gives the average aerosol yield per event (Figure 2c).

The count is a sum of the number of particles across all size bins (D_p_), which may also be shown as a size distribution (number of particles divided into size bins), illustrated in Figure 2d, expressed as particle number concentration (cm^-3^). Different sources of aerosol result in different particle sizes, each of which are referred to as a single mode. Mean aerosol yield and size distributions are extracted for each procedure per surgery and averaged over all surgeries to give overall aerosol yield and characteristic particle size distributions with standard errors, i.e. one surgery = one sample.

### Surgery

Patients were recruited from those attending routine or urgent orthopaedic surgery at Southmead Hospital, Bristol. Aerosol yield was recorded from 17 common orthopaedic open surgical procedures on the hip, knee, or shoulder, as summarized in Table I.

**Table I.** List of the 17 open orthopaedic surgeries during which aerosol yield was recorded: nine knee, two shoulder, and six hip operations.

Open orthopaedic procedures list (n = 17)
**Open knee (n = 9)**
Revision knee arthroplasty (cemented)
Multi-ligament reconstruction (open lateral and posterolateral corner reconstruction)
Hamstring harvest for ACL reconstruction
Secondary patella resurfacing and liner change in a TKA
Through knee amputation
TKA ( four cases – all cemented)
**Open shoulder (n = 2)**
Shoulder arthroplasty (two uncemented)
**Open hip (n = 6)**
Hip extracapsular trochanteric fracture fixation with plate
Revision hip arthroplasty (cemented)
THA (three cemented, one uncemented)

ACL, anterior cruciate ligament; THA, total hip arthroplasty; TKA, total knee arthroplasty.

Control measurements, where aerosol was measured during phantom procedures without a patient, were performed pre-surgery by recording 30 s instrument use in air, in triplicate. A phantom surgery was simulated, repetitively performing surgical movements with the lead surgeon wearing full aseptic precaution fluid-resistant orthopaedic grade disposable sterile surgical gown, and the non-patient volunteer with bare leg through drapes. All controls were performed under laminar flow, enabling identification of aerosol generated by non-patient sources (e.g. instruments, gown rubbing).

## Results

### Aerosol yield

**Fig. 3:**
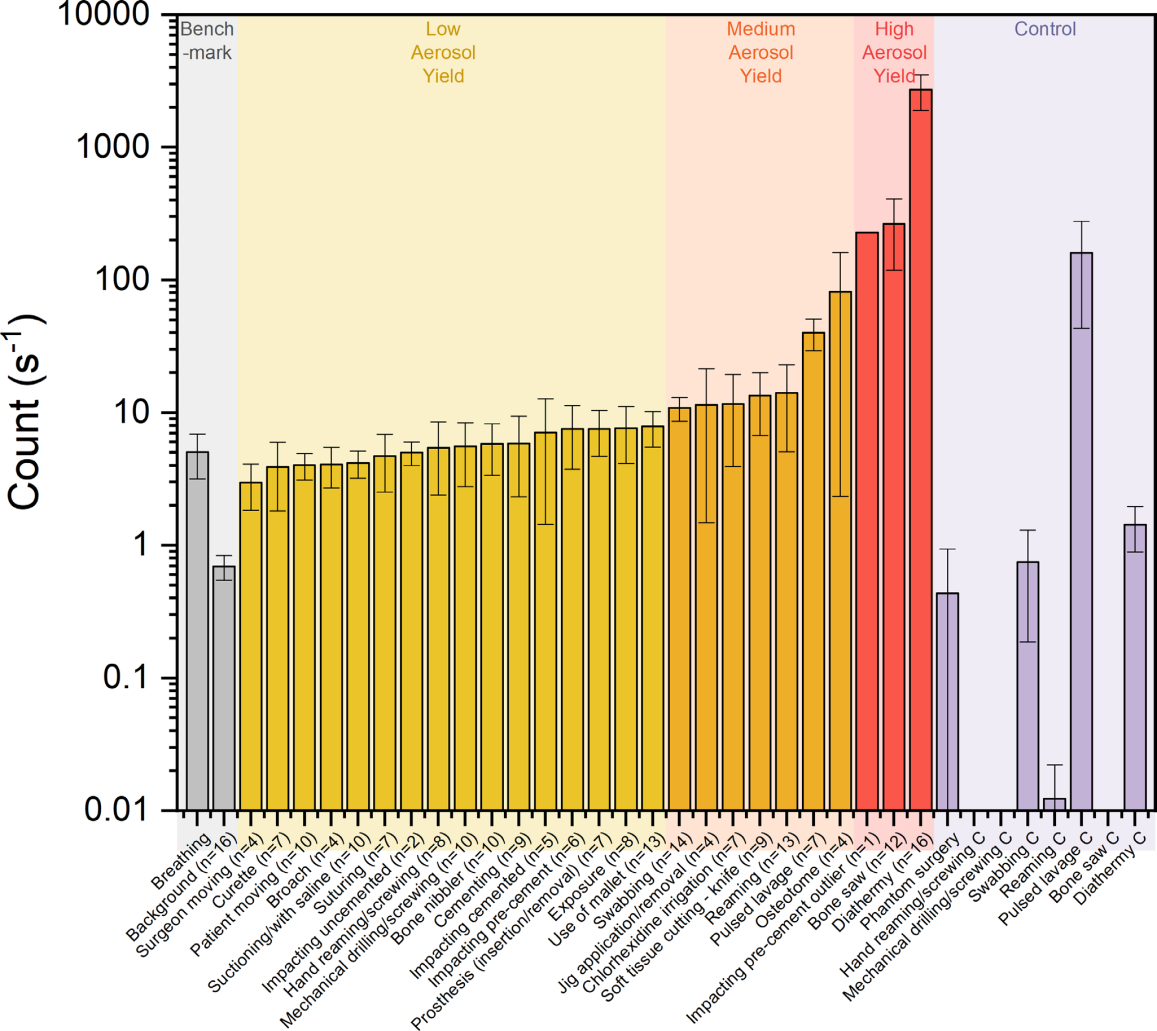
Mean aerosol yield of procedures detected at the site of the lead surgeon, averaged over 17 orthopaedic surgeries. Aerosol yield is categorized as: low, 1 to 10 s^-1^ (yellow); medium, 10 to 100 s^-1^ (orange); and high, > 100 s^-1^ (red). Aerosol yield from control (C) experiments (purple), mechanical drilling/screwing, hand reaming/screwing, and bone sawing were 0 s^-1^. Comparative benchmarks (grey) of breathing recorded at 20 cm^11^ and mean background. Standard error is shown.

Figure 3 shows the mean aerosol yield (count (s^-1^)) for each procedure across each surgery (one surgery = one sample). For reference, aerosol yield during breathing (5 s^-1^) is also shown (grey, data from Shrimpton et al^11^). These breathing data were recorded under laminar flow from the tidal breathing of 11 patients at 20 cm from the patient using an optical particle sizer (OPS); number concentrations are typically approximately 50% higher than reported by an APS, owing to the lower OPS detection size limit (0.3 μm).

Orthopaedic procedures are defined as: low, 1 to 10 s^-1^ (yellow); medium, 10 to 100 s^-1^ (orange); and high, > 100 s^-1^ (red) aerosol yield. Most notable are the high aerosol yield procedures diathermy (2,717 s^-1^) and bone sawing (265 s^-1^), alongside a single impacting of trial prosthesis pre-cement event (228 s^-1^). This procedure was separated as an outlier, given it had an aerosol yield order of magnitude greater than the other impacting procedures (5 to 8 s^-1^). Osteotome (82 s^-1^) and pulsed lavage (40 s^-1^) had a medium aerosol yield, whereas reaming (14 s^-1^), soft-tissue cutting (13.4 s^-1^), chlorhexidine irrigation (jug or syringe) (11.7 s^-1^), jig application/removal (12 s^-1^), and swabbing (10.8 s^-1^) were on the medium-to-low aerosol yield border. The remaining procedures were low-yield.

The mean background aerosol detected during orthopaedic surgery was 0.7 s^-1^ (grey), owing to these procedures being conducted in a laminar flow operating theatre, which has virtually no background aerosol in the APS size ranges.

Figure 3 (purple) also reports results from the control experiments and phantom operation. A high aerosol yield was detected from the pulsed lavage control (160 s^-1^), performed with saline into a receiver. Very low aerosol yields were detected for phantom surgery, rubbing swabs on plastic and diathermy in air (< 1.5 s^-1^). Reaming, mechanical drilling/screwing, hand reaming/screwing, and bone sawing in air did not yield detectable aerosol.

### Aerosol size distributions

**Fig. 4:**
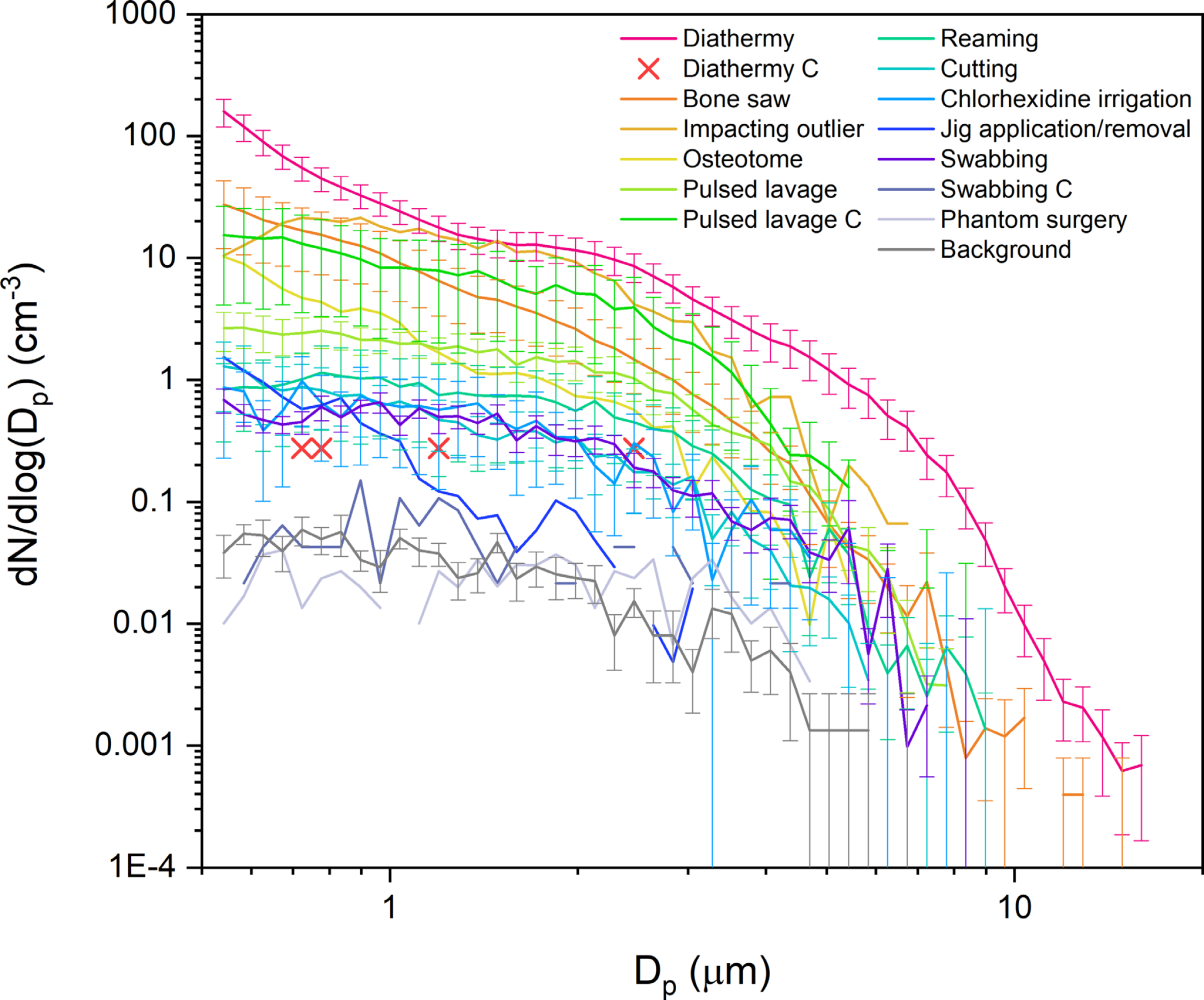
Size distributions for high and medium aerosol yield procedures from open orthopaedic surgery along with diathermy, pulsed lavage, swabbing, and controls (C) performed in air, phantom surgery, and background. Standard errors of the mean for osteotome, jig, and controls (excluding pulsed lavage) are large (not shown here). D_p_, size bin.

Aerosol size distributions can be unique fingerprints for aerosol sources and provide key information necessary to estimate the amount of aerosol mass generated, which is an important aspect of estimating risk. Mean aerosol particle size distributions from the high- and medium-yield procedures are shown in Figure 4, compared with those generated from the background and control experiments. The low-yield procedure size distributions are not displayed because the number of detected particles was too low to interpret the size distribution confidently.^10^

In Figure 4, diathermy (pink) generates a broad multimodal size distribution that, uniquely, indicates a substantial number of particles at sizes < 1 μm and extending out beyond 5 μm. The other procedures generate broad but less distinct size distributions that predominantly consist of particles ≤ 5 µm. Notably, both pulsed lavage (light green) and the associated control experiment (medium green) have similar size distributions.

### Duration of aerosol yield

An additional essential parameter to assess aerosol generation is the fraction of time aerosol was detected during a specific activity. Table II summarizes the number of surgeries in which the specific procedure was performed (n), the total number of seconds across all surgeries for which the procedure was in use, and the percentage of time that aerosol was detected above background. Aerosol from seven procedures was rarely detected (< 33%). Aerosol from five procedures was consistently detected (> 66%). Aerosol from the remaining majority of procedures was moderately frequently detected (33% to 66%). Note that the percentage for diathermy is > 100% because of the slow decay of aerosol after the procedure ended.

**Table II.** List of procedures with the total time (*t*) in seconds the procedure occurred, and number of surgeries in which the procedure was performed (n).

Procedure recorded (time (s), number of surgeries)	Percentage of time aerosol detected above background, %
Suturing (t = 3,110, n = 9)	10
Chlorhexidine irrigation (t = 894, n = 9)	14
Soft-tissue cutting – knife (t = 1,436, n = 12)	19
Impacting uncemented implants (t = 190, n = 3)	21
Suctioning/with saline (t = 765, n = 13)	24
Mechanical drilling/screwing (t = 846, n = 10)	30
Jig application/removal (t = 344, n = 4)	33
Cementing (t = 319, n = 9)	36
Hand reaming/screwing (t = 696, n = 10)	38
Reaming (t = 1,137, n = 13)	39
Exposure (t = 836, n = 8)	41
Bone nibbler (t = 481, n = 10)	41
Broach (t = 238, n = 4)	42
Use of mallet (t = 1,519, n = 15)	43
Curette (t = 498, n = 7)	44
Osteotome (t = 179, n = 4)	46
Impacting trial pre-cement (t = 285, n = 6)	51
Patient moving (t = 506, n = 10)	51
Prosthesis (insertion/removal trial) (t = 386, n = 7)	55
Surgeon moving (t = 364, n = 4)	56
Swabbing (t = 1,039, n = 14)	66
Impacting cemented implant (t = 72, n = 5)	69
Pulsed lavage (t = 779, n = 8)	79
Bone saw (t = 1,923, n = 12)	82
Diathermy (t = 3,444, n = 16)	126

## Discussion

In this study, we measured aerosol yield at the position of the surgeon 20 to 50 cm from the surgical site where aerosol is generated, allowing a relative assessment of the exposure risk. Aerosol yield from 25 distinct procedures was detected above background levels. Many low aerosol yield procedures generated aerosol yields comparable to those from breathing at a distance of 20 cm. From instrument use in air, diathermy was of minimal aerosol yield and the remaining (including phantom surgery) were indistinguishable from the background, except for pulsed lavage. Therefore, the aerosol detected from nearly all procedures arises largely from the patient and therefore has a biological origin. It has been suggested that smaller respiratory aerosols (≤ 5 µm) contain more RNA copies of SARS-CoV-2 than larger particles. This, coupled with enhanced respiratory penetration below this threshold, indicates that this is the high-risk aerosol size range.^12^ However, importantly, orthopaedically generated aerosols are certainly likely to contain substantially less viable virus than those generated by breathing or surgical procedures involving respiratory or mucosal systems, even compared with the higher aerosol yields discussed here.

### High aerosol yield

Diathermy was the highest aerosol yield by far (> 2,500 s^-1^) and was continuously detected at the site of the lead surgeon when in use. Consequently, diathermy likely presents an important health risk to clinicians, even as recent studies have suggested that viable SARS-CoV-2 is unlikely to survive the high-temperature process,^13^ given diathermy aerosol is known to contain carcinogens.^14^ Since aerosol generation from diathermy is well documented, smoke evacuation or N95 masks are already recommended for protection against inhalation of harmful smoke particles.^13,14^ Oscillating bone saw was also high aerosol yield (> 250 s^-1^), which was nearly continuously detected while in use. Previously, large quantities of aerosol generation from oscillating saws and contamination of the operating theatre have been found, and extra PPE has been recommended.^15,16^ The relative exposure of the lead surgeon compared with medium or low aerosol yield procedures is evident. However, these values cannot be used to define aerosol generation at source due to these procedures nor the expected viral load in the particles.

### Medium aerosol yield

Pulsed lavage is commonly listed as an AGP and has been proven to contaminate an operating theatre.^6,17^ Pulsed lavage was medium aerosol yield, which was consistently detected, but comparing the yield and size distributions of control and patient measurements strongly suggests that a substantial portion of the aerosol generated from pulsed lavage does not arise from the patient, but instead from the sterile saline solution applied to the surgical site through the lavage gun.

An advisory paper suggests that heavy instrument procedures are moderate-risk and should be used with care to avoid splashes, but there is no supporting literature.^6^ Yeh et al^18^ performed experiments in which heavy instrument procedures were used, but did not report the extent of specific aerosol yield per procedure. However, they indicated that use of a heavy tool (e.g. hammer) may generate aerosol. In this study, we showed that osteotome use and jig application/removal were medium aerosol yield and moderately frequently detected. It should be noted that osteotomes are generally used for short periods of time (e.g. only 179 s across six surgeries). The use of other heavy instruments (bone nibbler, hand reaming/screwing, and broach) was low-yield.

### Borderline medium-to-low/low aerosol yield

At the onset of the pandemic, all high-speed mechanical orthopaedic instruments were widely considered AGPs. However, this study found that mechanical drilling/screwing and mechanical reaming were borderline medium-to-low/low aerosol yield (5 to 14 s^-1^) and not consistently detected. Mechanical drilling has also been observed to have lower than expected drill splatter of larger droplets.^19^ This highlights that there are important distinctions to be made in the nature of the high-speed instrument. This difference is most likely due to the point of use – deep procedures inhibiting aerosol detection at the position of the surgeon. For instance, oscillating bone sawing is typically performed on an exposed bone, to minimize damage to adjacent structures, whereas reaming is deeper – e.g. reaming an acetabular component in total hip arthroplasty (THA) or within a bone intramedullary canal for revision THA/total knee arthroplasty (TKA) – and drilling/screwing is normally deep inside the body.

Low aerosol yield was obtained for impacting and use of mallet, but varied in consistency. A single impaction of trial pre-cement outlier may be the result of a particular surgeon’s technique and surgical site, and we suggest this should be treated as an exception – perhaps there was fluid overlying this trial implant that was not recorded. Alternatively, impaction aerosol generation may be directional relative to the striking angle, so is frequently undetected.

Aerosol generation from light, low-speed instrument procedures has not previously been considered. Here, we show that some border medium-to-low aerosol yield procedures (10 to 13 s^-1^) (swabbing, cutting, and chlorhexidine irrigation) rarely generate sufficient aerosol to be detected. The majority of these procedures were low aerosol yield (exposure, prosthesis (insertion/removal), cementing, patient or surgeon moving, and curettage).

Suctioning with and without saline was described as a key contributor to aerosol generation throughout the surgical procedure,^18^ however we found it to be low aerosol yield. It may be that suctioning/saline techniques have improved since the previous study was published in 1995.

### Further discussion

Here, a mixture of blood, tissue debris, bone dust, and irrigation fluid is aerosolized. SARS-CoV-2 acts by binding to angiotensin-converting enzyme 2 (ACE-2), which is expressed abundantly in the lungs and intestines,^20^ and less abundantly in blood, bone marrow, and muscle.^21^ SARS-CoV-2 viral RNA has been detected in the blood samples of COVID-19-positive patients, so it is possible that transmission may occur via aerosolized blood inhalation and subsequent binding to the ACE-2 receptor. However, currently there is no evidence for infectious virus in blood samples.^22^ The presence of SARS-CoV-2 RNA in bone or tissue remains undetermined. Beyond COVID-19, the long-term health risks associated with inhalation of aerosols produced during surgery, including surgical smoke, remain an important consideration for surgical teams,^23,24^ and the use of mitigation such as suction systems seems very reasonable.

In conclusion, during routine open orthopaedic surgery many procedures result in substantial aerosol signal detected at the site of the lead surgeon. Given that the lead surgeon often moves away from the site of surgery, these values are underestimates of total aerosol generation. Diathermy most notably, and bone sawing, were highly aerosol-generating. Care should be taken when performing these procedures. Smoke evacuation may be used for diathermy, and bursts rather than continuous use should be favoured if possible to allow laminar flow to effectively clear the environmental aerosol. Pulsed lavage generates aerosol, but this aerosol can be explained largely by instilled saline running through the instrument rather than patient-derived material. We find that, contrary to the previous broad classifications, AGPs cannot be classified based on factors such as high-speed mechanical action. Although the potential for SARS-CoV-2 transmission from aerosol generated during orthopaedic procedures is unclear, this study more broadly identifies procedures that pose a general health risk owing to high levels of surgically generated aerosol including biological aerosols and surgical smoke.
